# Physical activity, mental and physical health during the Covid-19 outbreak: longitudinal predictors of suicide ideation in Germany

**DOI:** 10.1007/s10389-022-01708-0

**Published:** 2022-03-25

**Authors:** Julia Brailovskaia, Inga Truskauskaite-Kuneviciene, Evaldas Kazlauskas, Odeta Gelezelyte, Tobias Teismann, Jürgen Margraf

**Affiliations:** 1 Mental Health Research and Treatment Center, Department of Clinical Psychology and Psychotherapy, Ruhr-Universität Bochum, Massenbergstr. 9-13, 44787 Bochum, Germany; 2 Center for Psychotraumatology, Institute of Psychology, Vilnius University, Vilnius, Lithuania

**Keywords:** Suicide ideation, Mental health, Physical health, Physical activity, Covid-19

## Abstract

**Aim:**

Suicide ideation has increased since the outbreak of Covid-19 in many countries. The present longitudinal study investigated potential predictors of suicide ideation.

**Subject and methods:**

Data of 406 participants from Germany (age *M* = 27.69, *SD* = 6.88) were assessed via online surveys in spring 2020 (baseline, BL) and in spring 2021 (follow-up, FU).

**Results:**

The current results reveal a significant increase in symptoms of depression, anxiety, and stress between 2020 and 2021. Positive mental health (PMH), sense of control, and physical health significantly decreased. Depression symptoms (BL), PMH (BL), and consciously enhanced physical activity since the pandemic outbreak (FU) significantly predicted 12-month suicide ideation (FU). In a moderated mediation analysis, the positive relationship between depression and suicide ideation was significantly mediated by PMH. Consciously enhanced physical activity significantly moderated the negative association between PMH and suicide ideation.

**Conclusion:**

The context of Covid-19 could negatively impact mental health and physical health. This might increase the risk for suicide ideation. However, PMH and physical activity might serve as protective factors. The protective effect of physical activity could be especially important in people with high depression symptoms and low PMH, such as clinical patients. Potential ways of how PMH and physical activity could be enhanced in the Covid-19 context to prevent suicide ideation are discussed.

## Introduction

Suicide is a leading cause of death among young people (Brunier and Drysdale 2021). Suicide ideation is a strong predictor of suicide attempts (May and Klonsky 2016). Recent research from different countries reported an increase in suicide ideation since the outbreak of the coronavirus disease (Covid-19; severe acute respiratory syndrome coronavirus 2, SARS-CoV-2) (e.g., Tanaka and Okamoto 2021; Killgore et al. 2020a). Young individuals are especially affected by this negative development (Cheung et al. 2021; O'Connor et al. 2020; Czeisler et al. 2020; Gelezelyte et al. 2021; Brailovskaia et al. 2021c). Against these findings and considering the uncertainty about the duration of the pandemic, it is important to identify factors that can predict suicide ideation in the context of Covid-19. This knowledge can be used to protect people at risk for suicide-related outcomes (suicide ideation, suicide attempts).

The experience of emotional stress and a low level of mental health can foster suicide-related outcomes (Smith et al. 2006). The Covid-19 outbreak and its restrictive consequences for everyday life are emotionally challenging (Klomek 2020). Social isolation, domestic violence, unemployment, health, and financial problems that resulted from the pandemic could negatively impact mental health (Reger et al. 2020; Peretti-Watel et al. 2020), which in consequence might foster an increase in suicide ideation (Tasnim et al. 2020). In line with this assumption, suicide ideation during Covid-19 was positively linked to symptoms of depression, anxiety, and stress (Killgore et al. 2020b; Caballero-Domínguez et al. 2020). Thus, an increase in depression, anxiety, and stress symptoms since the Covid-19 outbreak might at least partly explain the increase of suicide ideation during the pandemic.

However, not all people who experience negative symptoms have high levels of suicide ideation. Therefore, protective factors are assumed to reduce the risk for suicide ideation in the Covid-19 situation. Following recent results (Tasnim et al. 2020), one of such factors might be physical activity. In a cross-national study, regular physical activity (e.g., jogging, cycling, or yoga) buffered the impact of depression symptoms on the experience of psychological burden by the Covid-19 situation (Brailovskaia et al. 2021a). In addition to the benefits with respect to physical health (Vuillemin et al. 2005), physical activity and the achievement of small self-determined goals (e.g., increase of own jogging speed) can improve one’s mood and increase one’s sense of control (Rebar et al. 2015). Sense of control is an important human need (Skaff 2007). Individuals with a low sense of control often have enhanced levels of depression and anxiety (Keeton et al. 2008) and tend to make use of dysfunctional coping strategies such as helplessness and frustration in stressful situations (Southwick and Southwick 2018). In studies conducted prior to the onset of the pandemic, sense of control reduced suicide-related outcomes (Zhang et al. 2012; Dombrovski et al. 2018). Furthermore, physical activity and sense of control are positively linked to positive mental health (PMH)—that is, emotional, social, and psychological well-being (Lukat et al. 2016)—which is a well-known protective factor against suicide ideation and suicidal behavior (Teismann et al. 2019; Brailovskaia et al. 2020a). PMH confers resilience and contributes to functional coping strategies in uncertain and unexpected situations (Truskauskaite-Kuneviciene et al. 2020). Thus, people who consciously engage in regular physical activity during the pandemic might be at a lesser risk for suicide ideation. Further protective factors might be sense of control and PMH.

Available literature described a decrease in mental health since the pandemic outbreak (e.g., Bueno-Notivol et al. 2020). However, most of the research had a cross-sectional study design or compared the mental health data over a short period of time (e.g., during spring 2020) (Xiong et al. 2020). Thus, it remains unclear whether a significant long-term change of mental health variables can be attributed to the pandemic. Therefore, the first aim of our study was to compare the level of mental health and further potential predictors of suicide ideation over a follow-up period of one year (baseline, BL: spring 2020 vs. follow-up, FU: spring 2021). We hypothesized that symptoms of depression, anxiety, and stress increased during the past year (Hypothesis 1a). In contrast, we expected that the level of PMH and sense of control decreased during the past year (Hypothesis 1b). Moreover, the level of physical health can also influence suicide-related outcomes (Heisel and Flett 2008). Therefore, we included physical health in our investigation. Following previous research (Maugeri et al. 2020), we assumed a decrease in physical health during the past year (Hypothesis 1c).

The second aim of our study was to investigate potential factors and mechanisms that predict suicide ideation during the pandemic (i.e., during the past year). Available literature mainly focused on risk factors that could increase suicide ideation in the context of Covid-19, such as depression, anxiety, and stress (Caballero-Domínguez et al. 2020; Killgore et al. 2020b). However, it remains unclear whether protective factors identified before the pandemic outbreak, such as PMH (Teismann et al. 2018), remain effective in the context of Covid-19. Against this background, we hypothesized that symptoms of depression, anxiety, and stress at BL positively predict the 12-month suicide ideation assessed at FU (Hypothesis 2a). Furthermore, we assumed that PMH at BL (Hypothesis 2b), sense of control at BL (Hypothesis 2c), physical health at BL (Hypothesis 2d), and consciously increased physical activity at FU compared to the time before the Covid-19 outbreak (Hypothesis 2e) negatively predict the 12-month suicide ideation assessed at FU. Note, we considered consciously enhanced engagement in physical activity during the past year compared to the time before the Covide-19 outbreak as a protective factor against suicide ideation. Against this framework, we assessed the conscious increase of physical activity since the pandemic outbreak only at FU. Thus, a comparison of the level of physical activity between BL and FU was not possible.

**Fig. 1:**
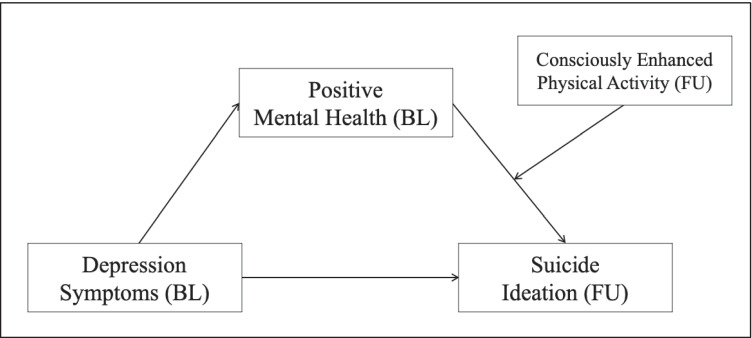
Moderated mediation model with depression symptoms (BL) (predictor), positive mental health (BL) (mediator), consciously enhanced physical activity (FU) (moderator), and suicide ideation (FU) (outcome). BL, Baseline; FU, Follow-Up

Siegmann et al. (2017) reported that individuals with enhanced levels of depression symptoms were at a specific risk for suicide ideation. PMH served as a protective factor and buffered this negative effect. To investigate the stability of this effect in the Covid-19 situation, we hypothesized that PMH at BL mediates the negative relationship between depression symptoms at BL and suicide ideation at FU (Hypothesis 3a). Furthermore, recent research described the moderating effect of physical activity on the experience of psychological burden by the Covid-19 situation. Individuals who engaged in physical activity experienced the pandemic outbreak and its consequences as less burdensome than other people (Brailovskaia et al. 2021a). Therefore, we assumed that a conscious increase in physical activity during the past year (assessed at FU) interacts with PMH at BL and thus moderates the association between PMH at BL and suicide ideation at FU (Hypothesis 3b). Specifically, the higher the level of physical activity, the stronger the protective effect of PMH on suicide ideation. Figure 1 illustrates the hypothesized associations as a moderated mediation model (Hayes 2021).

## Materials and methods

### Procedure and participants

In March 2020, a collective e-mail invitation to the first online survey (baseline, BL) was sent to 450 current or former students at a large university in the Ruhr region of Germany who had agreed to be contacted for research investigations. In March 2021, an e-mail invitation for the second survey (follow-up, FU) was sent to the 423 individuals who completed the first survey. Participation was voluntary and compensated by course credits for students. There were no specific requirements for participation. The final sample included 406 people who participated in both surveys (BL 75.1% women; age *M* = 27.69, *SD* = 6.88, range 18–71). At BL, 58.9% of the participants were students, 39.4% were employed, 1.5% were unemployed, and one person was retired; at FU, 51% were students, 47.3% were employed, 1.5% were unemployed, and one person was retired. Participants were instructed and gave informed consent to participate via an online form. The study’s implementation was approved by the responsible Ethics Committee. Power analyses using the G*Power program, version 3.1, indicated that of all analyses in the present study the moderated mediation analysis required the largest sample size of at least *N* = 311 for valid results (power > .80, *α* = .05, effect size: *f*^2^ = .02; cf., Mayr et al. 2007). Thus, the current sample size was sufficient.

### Measures

#### Depression, anxiety, and stress symptoms

The Depression Anxiety Stress Scales 21 (DASS-21; Lovibond and Lovibond 1995) measured symptoms of depression, anxiety, and stress with, respectively, seven items per subscale (e.g., depression subscale: “I felt that life was meaningless”; anxiety subscale: “I felt scared without any good reason”; stress subscale: “I found it hard to wind down”). All items are rated on a 4-point Likert-type scale (0 = *did not apply to me at all*, 3 = *applies to me very much or most of the time*). Higher sum scores indicate higher negative symptoms. Current scale reliability: depression: Cronbach’s *α*_*BL*_ = .905, *α*_*FU*_ = .926; anxiety: *α*_*BL*_ = .846, *α*_*FU*_ = .920; stress: *α*_*BL*_ = .881, *α*_*FU*_ = .906.

#### Positive mental health (PMH)

The unidimensional Positive Mental Health Scale (PMH-Scale; Lukat et al. 2016) assessed PMH. The nine items are rated on a 4-point Likert-type scale (e.g., “I enjoy my life”; 0 = *do not agree*, 3 = *agree*). The higher the sum score, the higher the level of PMH. Current scale reliability: *α*_*BL*_ = .927, *α*_*FU*_ = .935.

#### Sense of control

Following Niemeyer et al. (2019), the two-item scale (Item 1: “Do you experience important areas of your life (i.e., work, free-time, family, etc.) to be uncontrollable, meaning that you cannot, or barely can influence them?”; Item 2: “Do you experience these important areas of your life as unpredictable or inscrutable?”) was used to measure sense of control. Both items are rated on a 5-point Likert-type scale (0 = *not at all*, 4 = *very strong*). The higher the sum scores, the lower the sense of control. Current scale reliability: *α*_*BL*_ = .827, *α*_*FU*_ = .878.

#### Physical health

Participants’ current physical health status was assessed with the EuroQuol Visual Analogue Scale (EQ VAS; The Euroqol Group 2013). The visual analogue scale ranges from 0 (*worst imaginable health state*) to 100 (*best imaginable health state*). Higher scores reveal higher levels of physical health. Previous research demonstrated the validity of this short instrument (Janssen et al. 2013). In the current study, the mean test-retest reliability between BL and FU was *r*_mtrr_ = .510.

#### Consciously enhanced physical activity

To measure consciously enhanced physical activity since the Covid-19 outbreak, participants rated their agreement with the statement “Compared to the time before the Covid-19 outbreak, I consciously do more physical activity (e.g., jogging, cycling, yoga)” on a 7-point Likert-type scale (1 = *I totally disagree*, 7 = *I totally agree*) at FU. This statement was formulated for the present study by the authors.

#### Suicide ideation

The 12-month suicide ideation was assessed with the relevant item (“How often have you thought about killing yourself in the past year?”) of the Suicide Behaviors Questionnaire – Revised (SBQ-R; Osman et al. 2001) at FU. The item is rated on a 5-point Likert-type scale (1 = *never*, 5 = *very often*). Twelve-month suicide ideation is revealed for a SBQ-R score > 1. The use of this instrument for screening purpose in clinical and nonclinical samples is well-established (Brailovskaia et al. 2020b).

### Statistical analyses

Statistical analyses were conducted using SPSS 26 (IBM Corp. 2019) and the macro Process version 3.5 (www.processmacro.org/index.html; Hayes 2021). After descriptive analyses, the levels of depression, anxiety, and stress symptoms, PMH, sense of control, and physical health were compared between BL and FU by repeated measures analyses of variance (within-subjects ANOVAs). The Greenhouse-Geisser correction (ε) was applied for all variables because of the violation of the assumption of sphericity. Partial eta-squared (η^2^_p_) was used as the effect-size measure. All post-hoc comparisons were Bonferroni-corrected (level of significance: *p *< .05, two-tailed).

Next, a two-step hierarchical regression analysis was calculated that included 12-month suicide ideation (FU) as the outcome. Step 1 included gender (BL; coded 0 = *woman*, 1 = *man*) and age (BL) as control variables; symptoms of depression, anxiety, and stress (all BL), PMH (BL), sense of control (BL), physical health (BL), and consciously enhanced physical activity (FU) were added in Step 2. There was no violation of the multi-collinearity assumption (all values of tolerance > .25, all variance inflation factor values < 5; Urban and Mayerl 2006).

Then, a moderated mediation analysis was run (Process: model 14). The model included a conditional indirect effect (see Fig. 1) and examined the multiple effects simultaneously (integration of the hypothesized mediation and moderation effects) (Edwards and Lambert 2007; Hayes 2018). The bootstrapping procedure (10,000 samples) that provides percentile bootstrap confidence intervals (*CI* 95%) assessed the moderated mediation effect. Depression symptoms (BL) served as the predictor, PMH (BL) as the mediator, consciously enhanced physical activity (FU) as the moderator, and 12-month suicide ideation (FU) as the outcome; controlling for the covariates age (BL) and gender (BL). The relationship between depression symptoms (BL) and PMH (BL) was denoted by path *a*; path *b* denoted the link between PMH (BL) and suicide ideation (FU); the relationship between depression symptoms (BL) and suicide ideation (FU) after the inclusion of PMH (BL) and consciously enhanced physical activity (FU) in the model was denoted by path *c’* (the direct effect).

## Results

Twelve-month suicide ideation (SBQ-R suicide ideation >1) was found in 30% (*n* = 122) of the sample: rarely (one time): 13.3% (*n* = 54), sometimes (two times): 9.1% (*n* = 37), often (three to four times): 3.4% (*n* = 14), and very often (five or more times): 4.2% (*n* = 17). Table 1 shows the descriptive statistics of the investigated variables and the results of the within-subjects ANOVAs. The comparisons reveal a significant increase in depression, anxiety, and stress symptoms between BL and FU (all: *p* < .001 and small effect). The level of PMH (large effect), sense of control (small effect), and physical health (medium effect) significantly decreased between both measurement time points (all: *p* < .001).

**Table 1:** Descriptive statistics and repeated measures analyses of variance (ANOVAs) of investigated variables at baseline and follow-up

	BL	FU			
	*M (SD)*	*M (SD)*	F	*p*	η^2^_p_
Depression symptoms	4.73 (4.73)	6.00 (5.62)	20.311	< .001	.048
Anxiety symptoms	2.53 (3.56)	3.53 (5.04)	15.353	< .001	.037
Stress symptoms	6.40 (4.82)	7.50 (5.43)	15.353	< .001	.037
Positive mental health	18.62 (5.96)	16.28 (6.67)	74.842	< .001	.156
Sense of control	2.74 (2.00)	3.25 (2.22)	20.336	< .001	.048
Physical health	79.97 (14.24)	75.38 (17.66)	33.137	< .001	.076
Consciously enhanced physical activity		3.23 (2.07)			
Suicide ideation		1.59 (1.07)			

* N* = 406; *BL*, baseline, *FU*, follow-up; *M*, Mean, *SD*, Standard Deviation, *p* = significance, η^2^_p_ = effect size-measure; degrees of freedom of all F-values: 1,405; sense of control: higher scores indicate lower sense of control

Table 2 provides the results of the regression analysis. The overall model explained 21.5% of the variance. Only depression symptoms (BL, *p* < .001), PMH (BL, *p* < .001), and consciously enhanced physical activity (FU, *p* < .05) served as significant predictors of the 12-month suicide ideation (FU) (see Table 2). The significant association between depression symptoms (BL) and the 12-month suicide ideation (FU) was positive. In contrast, the significant association between PMH (BL) and the 12-month suicide ideation (FU), and the significant association between consciously enhanced physical activity (FU) and the 12-month suicide ideation (FU) were negative (see Table 2).

**Table 2:** Hierarchical regression analyses (outcome: suicide ideation at follow-up)

	ß	95% CI	T	Adjusted R^2^	Changes in R^2^
*Step 1, F(2,403) = .475, p = .622*				−.003	.002
Age (BL)	−.025	[−.019, .011]	−.499		
Gender (BL)	.044	[−.134, .350]	.877		
*Step 2, F(9,396) = 12.232, p < .001*				.200	.215
Age (BL)	−.002	[−.014, .013]	−.054		
Gender (BL)	.020	[−.169, .267]	.441		
Depression symptoms (BL)	.303**	[.036, .100]	4.152		
Anxiety symptoms (BL)	−.059	[−.055, .019]	−.940		
Stress symptoms (BL)	−.060	[−.043, .017]	−.870		
Positive mental health (BL)	−.281**	[−.073, −.028]	−4.368		
Sense of control (BL)	−.047	[−.082, .032]	−.856		
Physical health (BL)	−.010	[−.008, .007]	−.203		
Consciously enhanced physical activity (FU)	−.105*	[−.099, −.008]	−2.328		

*N* = 406; gender: 0 = woman, 1 = man; ß, standardized coefficient beta; *CI*, Confidence Interval; **p < .01, *p < .05

As shown in Table 3, the moderated mediation analysis revealed a significant overall model, *F*(6,399) = 12.129, *p* < .001. The explained variance of the overall model was *R*^*2*^ = .225. The direct effect (path *c’*) of depression symptoms (BL) on suicide ideation (FU) was significant (*p* = .002) after controlling for PMH (BL), consciously enhanced physical activity (FU), and their interaction. The conditional indirect effect of depression symptoms (BL) on suicide ideation (FU) through PMH (BL) was significant in people with low (that is, one standard deviation below the mean in the analysis = −2.072) and medium (that is, the mean in the analysis = 0) levels of consciously enhanced physical activity (FU) (effect: low > medium; see Table 3). In contrast, it was not significant in people with high levels (that is, one standard deviation above the mean in the analysis = 2.072) of consciously enhanced physical activity (FU). As indicated by the index of moderated mediation, the test of moderated mediation was significant, revealing a significant moderated mediation effect (see Table 3).

**Table 3:** Moderated mediation model (outcome: suicide ideation at follow-up)

	ß	SE	t	*p*	95% *CI*
Path *a*: Depression symptoms (BL) ➔ PMH (BL)	−.817	.056	−14.634	< .001	[−.927, −.708]
Path *b*: PMH (BL) ➔ Suicide ideation (FU)	−.044	.012	−3.748	< .001	[−.067, −.021]
Interaction: PMH (BL)*physical activity (FU) ➔ Suicide ideation (FU)	.011	.005	2.470	.014	[.002, .020]
Path *c’* (direct effect): Depression symptoms (BL) ➔ Suicide ideation (FU)	.046	.014	3.198	.002	[.018, .074]
*Conditional indirect effects: Depression symptoms (BL) ➔ Suicide ideation (FU)*					
Depression symptoms (BL) ➔ PMH (BL) ➔ Suicide ideation (FU)					
Conscious physical activity (BL):					
Low (one SD below mean = −2.072)	.055	.013			[.030, .079]
Medium (mean = 0)	.036	.009			[.017, .053]
High (one SD above mean = 2.072)	.017	.011			[−.006, .038]
*Index of moderated mediation*	−.009	.004			[−.017, −.002]

*N* = 406; covariates: age and gender; *PMH*, Positive mental health; Physical activity = consciously enhanced physical activity; *BL*, Baseline; *FU*, Follow-up; *ß*, Standardized Beta; *SE*, Standard error; *t* = *t*-test; *p* = significance; *CI*, Confidence interval; explained variance of the overall model: *R*^*2*^ = .225

## Discussion

Since the Covid-19 outbreak, suicide ideation has increased in different countries (e.g., Tanaka and Okamoto 2021). The present longitudinal study provides evidence for a decrease in mental health and physical health during the pandemic in Germany. Furthermore, it identified significant factors and mechanisms that could predict suicide ideation over a year after the onset of the pandemic.

The Covid-19 outbreak was accompanied by a decrease in mental health (Galea et al. 2020). Following the dual-factor models of mental health (e.g., Suldo and Shaffer 2008; Antaramian et al. 2010), mental health is not only the absence of psychopathology, but consists of two distinct but interrelated dimensions: negative mental health and positive mental health (Antaramian et al. 2010; Trompetter et al. 2017). Both dimensions should be considered when making reliable conclusions about people’s mental health (World Health Organization 2001). The present results show that both dimensions of mental health could be impacted since the Covid-19 outbreak. Symptoms of depression, anxiety, and stress that represent the negative dimension significantly increased between spring 2020 and spring 2021 (confirmation of Hypothesis 1a). This finding is in line with previous research (e.g., Bueno-Notivol et al. 2020; Pretorius 2021). The fight against Covid-19 resulted in significant and emotionally challenging changes in different areas of people’s lives, for example, social isolation, job loss, and permanent worry about one’s health and the health of close others, which could contribute to the increase of the negative symptoms (Klomek 2020; Bayın et al. 2021; Evans et al. 2021).

Moreover, we found a decrease in PMH that represents the positive dimension of mental health between 2020 and 2021 (confirmation of Hypothesis 1b). Notably, this change revealed the highest statistical effect in comparison to the other investigated variables. Positive emotions experienced in social interactions, a regular sleep rhythm, and a high sense of control belong to the positive predictors of PMH (e.g., Shaban et al. 2020; Peach et al. 2016; Barry 2009). Since the Covid-19 outbreak, due to enhanced homeschooling and working from home, face-to-face social interaction has been restricted (Lemenager et al. 2021), and the regularity of sleep rhythm—as well as the overall sleep hygiene—of many people decreased (Cellini et al. 2020). Furthermore, we found a decrease with respect to the individual sense of control in the present study (confirmation of Hypothesis 1b). These changes could at least partly explain the significant decrease in PMH between 2020 and 2021.

The current findings contribute to the assumption that the Covid-19 outbreak and its consequences not only negatively impacted mental health but could also result in a decrease of physical health (confirmation of Hypothesis 1c). Considering available literature, this might be directly due to the infection by the coronavirus but also to reduced sleep hygiene, increased tendency to less regular and less healthy eating behavior, and enhanced sedentary behavior since the pandemic outbreak (Bates et al. 2020; Evans et al. 2021). Moreover, because mental health and physical health are interrelated (Ohrnberger et al. 2017), the reduction of one of them could negatively impact the other one.

Our results revealed changes in various variables of mental and physical health during the past year. However, they also show that not all of these variables could longitudinally predict suicide ideation over one year after the onset of Covid-19. In line with longitudinal research conducted prior to the Covid-19 outbreak (Wenzel et al. 2011; Teismann et al. 2018), depression symptoms at BL served as a positive predictor of the 12-month suicide ideation assessed at FU (partly confirmation of Hypothesis 2a). Thus, individuals who had enhanced depression symptoms at the beginning of the pandemic in 2020 could be at enhanced risk for suicide ideation over the following year. In contrast, symptoms of anxiety and stress at BL did not predict the 12-month suicide ideation assessed at FU (partly contradiction of Hypothesis 2a). This finding corresponds to the findings of Ibrahim et al. (2014) who also used the DASS-21 for the assessment of the negative symptoms. The authors explained the insignificant results by the rather general character of the used anxiety and stress scales that might not be sensitive enough to assess the factettes of anxiety and stress possibly contributing to suicide ideation (Ibrahim et al. 2014). Notably, in recent studies, Covid-19 related anxiety and Covid-19 related stress positively predicted suicide ideation (Killgore et al. 2020b; Caballero-Domínguez et al. 2020). Therefore, the explanation of Ibrahim et al. (2014) could at least partly apply to our results.

In line with earlier research (Teismann et al. 2018), PMH at BL negatively predicted suicide ideation over a period of one year (confirmation of Hypothesis 2b). Individuals with a high level of PMH are optimistic and emotionally stable. They tend to make use of functional coping strategies in uncertain situations and have a high level of resilience (Truskauskaite-Kuneviciene et al. 2020). The current results reveal that the protective effect of PMH could prevail during the Covid-19 outbreak.

In contrast, sense of control at BL (contradiction of Hypothesis 2c) and physical health at BL (contradiction of Hypothesis 2d) did not predict suicide ideation over the following year. One might speculate that the insignificance is due to the specificity of the Covid-19 situation. The introduced governmental measures limit the sense of control in general for many people (Zhu et al. 2020), especially those who previously used to travel and meet others at work and in their leisure time (Brailovskaia and Margraf 2021). This could put the impact of sense of control on suicide ideation into perspective. Furthermore, the investigated sample included mostly physically healthy and young individuals, which might limit the significance of physical health for suicide ideation. Notably, research that described a close association between physical health and suicide-related outcomes mostly focused on older individuals with health problems (e.g., Heisel and Flett 2008). However, to prevent speculation, further investigations require a replication of the present findings in a more gender-balanced sample. The use of measures that assess different areas of physical health—not only the overall physical health state as in the current study—might provide further insights in this context.

As expected, a conscious enhancement of physical activity compared to the time before the Covid-19 outbreak negatively predicted suicide ideation over a period of one year (confirmation of Hypothesis 2e). Physical activity is an important protective factor of mental health and physical health (Rebar et al. 2015; Haskell et al. 2009). The World Health Organization (2020) recommends approximately 150 min of moderate physical activity throughout the week. Earlier research emphasized that regular engagement in physical activity can reduce the risk for suicide-related outcomes (Vancampfort et al. 2018). The present findings show that its protective effect could remain stable during the Covid-19 outbreak.

Thus, depression symptoms and PMH in 2020, as well as the consciously enhanced physical activity during the past year significantly predicted a person’s 12-month suicide ideation in 2021. The results of the moderated mediation model provide further insight into the relationship between the four variables. PMH at BL mediated the effect of depression symptoms at BL on the 12-month suicide ideation assessed at FU (confirmation of Hypothesis 3a). Furthermore, consciously enhanced physical activity assessed at FU moderated the association between PMH at BL and the 12-month suicide ideation assessed at FU (partly confirmation of Hypothesis 3b). However, the direction of the moderation effect was unexpected. The enhancement of physical activity did not directly increase the protective effect of PMH on suicide ideation (contradiction of Hypothesis 3b). Rather the closeness of the association between PMH and suicide ideation decreased with higher levels of consciously enhanced physical activity. These findings can be interpreted as follows: PMH is an important protective factor of suicide ideation during the Covid-19 pandemic. Nevertheless, a high level of depression at the beginning of the pandemic could contribute to a low PMH level and therefore increase the risk for suicide ideation during the pandemic. However, conscious enhancement of physical activity could reduce this negative effect and specifically protect individuals with high levels of depression. Engagement in physical activity fosters positive emotions that people with high levels of depression often lack (Eime et al. 2013; Harris et al. 2006). This could compensate for a low level of PMH. Thus, both PMH and consciously enhanced engagement in physical activity might reduce the risk for suicide ideation in the Covid-19 situation. Individuals with high PMH levels could be strong and resilient enough to cope with the negative consequences of the pandemic and not tend to suicide ideation. In contrast, people with a low level of PMH and a high level of depression symptoms—which are typical characteristics of clinical inpatients (Spasojević and Alloy 2001)—could significantly benefit from enhanced physical activity that might reduce their risk for suicide ideation over one year after the onset of the Covid-19 outbreak.

The present results reveal the need for official programs that focus on the increase of PMH and physical activity, especially in the Covid-19 situation. Both could reduce the risk for suicide ideation that has increased across borders since the pandemic outbreak (Tanaka and Okamoto 2021; Killgore et al. 2020a; Gelezelyte et al. 2021; O'Connor et al. 2021). Recent research described a decrease in physical activity in the past year (e.g., Caputo and Reichert 2020; Kontsevaya et al. 2021; Rogowska et al. 2020), which underlines the urgency for programs that could publicly promote activities such as gymnastics, yoga, and jogging that do not require expensive equipment and can be performed by maintaining social distancing. Public governmental communication about this issue could be complemented by online (e.g., social media) and offline (e.g., billboards) advertising campaigns. Moreover, individuals who are less motivated to engage in physical activity on their own might benefit from online group trainings via videotelephony (e.g., ZOOM, Skype). Promoting physical activity could contribute to the experience of the Covid-19 situation as less stressful, increase positive emotions, self-efficacy and self-esteem and could foster the level of PMH and reduce suicide ideation (Southerland et al. 2016; Richards et al. 2015; Brailovskaia et al. 2021a).

A further focus of the public programs might be on mindfulness training. Mindfulness describes the enhanced attention to and the nonjudgmental awareness of the present moment (Bishop et al. 2004). It is positively linked to the experience of positive emotions, the ability to cope with extraordinary situations—such as the Covid-19 outbreak—and to solve challenging problems (Perestelo-Perez et al. 2017; Gaiswinkler and Unterrainer 2016). In a recent study, a mindfulness training contributed to PMH (Dumarkaite et al. 2021). Thus, the promotion of online mindfulness training allowing social distancing could foster PMH (see e.g., Totzeck et al. 2020; Matiz et al. 2020) and therefore reduce suicide ideation.

The following limitations of the present study are to be considered. First, the present findings are drawn from a relatively young and mostly female sample from Germany. To investigate their universality, especially of the moderated mediation model, they should be replicated in more gender-balanced age groups in different countries, as well as with data assessed at further measurement time points (e.g., two years after the Covid-19 outbreak). Second, the 12-month suicide ideation was measured only at FU. Thus, no conclusions about the change in suicide ideation during the Covid-19 outbreak can be drawn. Third, we assessed the consciously enhanced engagement in physical activity compared to the time before the Covid-19 outbreak at FU with only one self-constructed item. Thus, no direct comparisons of the level of physical activity are possible. Also, no information about the type of physical activity was assessed. Therefore, it remains unclear whether the found effects of physical activity are common for physical activity in general or for specific activities (e.g., yoga or jogging). In addition, the item used to assess consciously enhanced engagement in physical activity was constructed for the present study. Thus, its validation is an important issue for future research. Furthermore, it is important to note that both the consciously enhanced engagement in physical activity and the suicide ideation were retrospectively assessed at FU for a 12-month period. Against this background, conclusions on the direction of the relationship between both variables that was investigated by Hypothesis 2e and Hypothesis 3b in the present study should be considered with caution. Fourth, the data were assessed by self-report measurements that are prone to social desirability and same-source bias (Musch et al. 2002; Conway and Lance 2010). Future studies that replicate the present findings should measure and include social desirability in the statistical analyses (e.g., Balanced Inventory of Social Desirability; Musch et al. 2002). Furthermore, they should include additional data sources, for example, objective measures of physical health. However, assessing such data during the pandemic is challenging due to the requirement for social distancing. Fifth, recent research reported a positive association between PMH and the level of adherence to the behavioral measures such as wearing of face masks and keeping of social distancing in public places that were introduced to slow down the pandemic spread. In contrast, depression and anxiety symptoms were negatively linked to the level of adherence (Lavallee et al. 2021). Thus, it could be hypothesized that adherence to the measures could foster the protective effect of PMH on suicide ideation, and that it could weaken the effect of depression symptoms on suicide ideation. Moreover, the willingness to receive Covid-19 vaccination was negatively associated with stress symptoms. The valence of the association between PMH and vaccination willingness was inconclusive. In some samples, it was positive. In other samples, it was negative (Brailovskaia et al. 2021b). Thus, the question raises whether and how vaccination willingness could influence the effect of PMH on suicide ideation. Being infected by Covid-19 and staying in domestic quarantine are further factors that can impact the assessed variables of mental health (Bates et al. 2020; Evans et al. 2021), which could also influence one’s suicide ideation. In the present study, no Covid-19 specific variables were assessed. Therefore, future studies should replicate the present findings by the inclusion of further factors that could influence them such as adherence to governmental measures, vaccination willingness, being infected by Covid-19 and staying in domestic quarantine.

In conclusion, the present study confirms an increase in depression, anxiety, and stress symptoms since the Covid-19 outbreak. In contrast, the level of positive mental health, sense of control, and physical health decreased. Furthermore, individuals with high levels of depression symptoms at the onset of the pandemic seem to be at enhanced risk for suicide ideation over one year. Positive mental health and consciously enhanced engagement in physical activity could reduce this risk. Public governmental communication should present ways to foster both protective factors while maintaining social distancing.

## Data Availability

The dataset and further material analyzed during the current study will be available from the corresponding author on reasonable request.

## Code availability (software application or custom code)

Statistical analyses were conducted using SPSS 26 and the macro Process version 3.5 (www.processmacro.org/index.html). The used statistical codes will be available from the corresponding author on reasonable request.
